# Angiotensin II receptor blocker telmisartan attenuates aortic stiffening and remodelling in STZ-diabetic rats

**DOI:** 10.1186/1758-5996-6-57

**Published:** 2014-05-20

**Authors:** Erik Salum, Mark Butlin, Jaak Kals, Mihkel Zilmer, Jaan Eha, Alberto P Avolio, Andres Arend, Marina Aunapuu, Priit Kampus

**Affiliations:** 1 Department of Cardiology, University of Tartu, 8 Puusepa Street, Tartu 51014, Estonia; 2 Endothelial Centre, University of Tartu, 8 Puusepa Street, Tartu 51014, Estonia; 3 Department of Biochemistry, Centre of Excellence for Translational Medicine, University of Tartu, 19 Ravila Street, Tartu 50411, Estonia; 4 The Australian School of Advanced Medicine, 2 Technology Place, Macquarie University, Sydney, NSW 2109, Australia; 5 Department of Vascular Surgery, Tartu University Hospital, 8 Puusepa Street, Tartu 51014, Estonia; 6 Department of Anatomy, University of Tartu, 19 Ravila Street, Tartu 50411, Estonia; 7 Institute of Veterinary Medicine and Animal Sciences, Estonian University of Life Sciences, 62 Fr. Kreutzwaldi Street, Tartu 51014, Estonia

**Keywords:** Arterial stiffness, Collagen, Diabetes, Elastin, Pulse wave velocity, Streptozotocin, Telmisartan

## Abstract

**Background:**

Prevention or attenuation of diabetic vascular complications includes anti-hypertensive treatment with renin-angiotensin system inhibitors on account of their protective effects beyond blood pressure reduction. The present study aimed to investigate the effects of telmisartan, an angiotensin II type 1 receptor blocker (ARB), on blood pressure, aortic stiffening, and aortic remodelling in experimental type 1 diabetes in rats.

**Methods:**

Diabetes was induced by streptozotocin (STZ) (65 mg/kg) in male Wistar rats. One diabetic group was treated for 10 weeks with telmisartan (10 mg/kg/day p/o). Pressure-independent aortic pulse wave velocity (PWV) was measured under anaesthesia after intravenous infusion of phenylephrine and nitroglycerine. Aortic wall samples were collected for histomorphometrical analysis.

**Results:**

Untreated diabetes imposed differential effects on aortic stiffening, as demonstrated by increased isobaric PWV over a range of high blood pressures, but not at lower blood pressures. This was associated with loss and disruption of elastin fibres and an increase in collagen fibres in the aortic media. Treatment with telmisartan decreased resting blood pressure, reduced aortic stiffness, and partially prevented the degradation of elastin network within the aortic wall.

**Conclusions:**

Telmisartan improved the structural and functional indices of aortic stiffening induced by untreated STZ-diabetes, demonstrating the importance of ARBs in the therapeutic approach to diabetic vascular complications.

## Background

Diabetes mellitus (DM) is a major cardiovascular (CV) risk factor, as evidenced by three-fold higher risk of developing CV complications in patients with DM [1]. Functional and structural changes in the large arteries are an important contributor to high mortality in the diabetic population [2]. Metabolic abnormalities associated with DM impair the integrity of elastic fibres in the arterial wall which translates into increased stiffness of the wall material. The accumulation of advanced glycation end-products (AGEs) is a major pathogenic mechanism contributing to arterial stiffening in DM [3]. Assessment of arterial stiffness by measuring pulse wave velocity (PWV) is a well-established method and large-scale studies have demonstrated its utility in predicting the CV risk and outcome in general population [4] and in high-risk conditions, including hypertension [5], end-stage renal disease [6], and DM [7,4].

The renin-angiotensin system (RAS) has an important role in the regulation of arterial structure and function. Angiotensin II (Ang II) induces vasoconstriction and has been shown to contribute to vascular wall remodelling [8] via non-haemodynamic mechanisms such as stimulation of vascular smooth muscle proliferation and rearrangements of elastin and collagen fibres [9]. The renin-angiotensin system activity in vascular tissue is known to be increased under hyperglycaemic conditions [10], promoting vascular hypertrophy and wall stiffness.

Blockade of RAS with either angiotensin-converting enzyme inhibitors or Ang II type 1 receptor blockers (ARBs) has been reported to improve CV outcomes and reduce the risk of micro- and macrovascular complications of DM [11-13]. Furthermore, clinical studies have demonstrated that while RAS blockers are equally effective in blood pressure (BP) reduction, they may be superior to other antihypertensive agents in reducing the risk of CV events in patients with DM [13,14]. It is suggested that the protective effects of RAS blockers on the vasculature are, at least in part, due to reduction in the adaptive remodelling and stiffness of the arterial wall independently on their BP lowering effects.

Among ARBs, telmisartan has exhibited beneficial metabolic effects by increasing insulin sensitivity [15] and improving plasma lipid profile [16]. These properties indicate that telmisartan may provide unique possibilities for the prevention and treatment of DM and its vascular complications [17].

Previously, we have demonstrated that treatment with vitamin D improves aortic wall remodelling induced by short-term uncontrolled experimental DM, but does not limit the development of aortic stiffening assessed by PWV under pressure-independent conditions [18]. The aim of the present study was to examine the hypothesis that telmisartan would attenuate the development of diabetes-induced aortic remodelling, and that this would be associated with reduction in aortic stiffness independently on BP.

## Materials and methods

### Animals

Male Wistar rats (RccHan:WIST, n = 40, age 3 weeks) were purchased from Harlan Laboratories (Harlan Laboratories, Inc., The Netherlands). Before entering the experiments, the animals were allowed to acclimatise for at least one week in the animal facility. All animals were kept in a room with controlled temperature (21 ± 2°C) and lighting (12:12-h light–dark cycle) with free access to food pellets and tap water. All experimental procedures were approved by the Estonian National Board of Animal Experiments and were conducted in accordance with the Directive 2010/63/EU of the European Parliament.

### Experimental protocol

Rats were randomly assigned to three groups: control (n = 10), untreated diabetic (n = 15), and telmisartan-treated diabetic (n = 15) group. Intraperitoneal injection of streptozotocin (STZ) 65 mg/kg (Sigma-Aldrich, St. Louis, MO, USA), freshly dissolved in 0.9% saline, was used to induce diabetes. After 48 hours, blood glucose was measured in samples taken from the tail vein, using a glucometer (Glucocard X-meter, Arkray Inc., Japan). Rats were considered diabetic if the blood glucose was >15 mmol/L. Treatment with telmisartan (Boehringer Ingelheim International GmbH, Germany) was started immediately after confirmation of diabetes. Telmisartan (10 mg/kg per day) was administered by gavage, dissolved in drinking water, for 10 weeks. Telmisartan was stopped one day prior to measurement of the haemodynamic parameters. Weekly, body weights were recorded for all groups and glycosuria was assessed with reagent strips (Combur Test, Roche, Germany) to exclude ketosis in rats with diabetes.

### Haemodynamic measurements

The animals were anaesthetised with a mixture of fentanyl (0.07 mg/kg, Gedeon-Richter Plc., Hungary), midazolam (5 mg/kg, Roche Pharma AG, Germany), and ketamine (75 mg/kg Vetoquinol Biowet Sp. z.o.o., Poland) administered subcutaneously. The optimal concentrations of the anaesthetic substances had been determined in previous experiments [18]. The depth of anaesthesia was monitored regularly by assessing the reflex response to noxious stimuli (hindpaw pinch) or tactile stimuli (corneal stroking). After induction of anaesthesia, animals were placed on a heating pad and body temperature was maintained at 37°C.

A 2.5 F high-fidelity, dual pressure sensor catheter with 50 mm separation between sensors (SPC-721, Millar Instruments Inc., TX, USA) was soaked in a water bath for 30 minutes to allow stability of the baseline before balancing and calibration against a mercury sphygmomanometer. The catheter was then introduced via the femoral artery into the descending aortic trunk so that the distal sensor was positioned at the beginning of the descending aorta and the resulting position of the proximal sensor was just proximal to the aortic bifurcation. The positioning of the distal sensor in the thoracic aorta was confirmed by the appearance of the dicrotic notch on the pressure waveform. The rats were allowed to stabilise before resting blood pressures were recorded. Mean arterial pressure (MAP) was determined from measurements made by the proximal pressure transducer. Mean arterial pressure was increased and decreased by infusion of phenylephrine (PE) (50 μg/min) and nitroglycerine (NTG) (30 μg/min), respectively, via a catheter inserted into the femoral vein. Each drug was infused until the BP plateaued. Between the infusions, the rats were allowed a stabilisation period of at least 5 minutes and subsequent infusion of the opposing substance was given after the BP had returned to baseline. Pulse pressure waves were recorded simultaneously at the two aortic sites and PWV was calculated offline by dividing the propagation distance by propagation time using an automated foot-to-foot method [18,19]. Data were acquired at a sampling rate of 2 kHz (PowerLab, ADInstruments, Australia) and feature extraction and calculations made with custom scripts in Spike2 v.6. software (Cambridge Electronic Design, United Kingdom).

### Laboratory parameters

After completion of haemodynamic measurements, rats were euthanised by cardiac puncture and cervical dislocation. Urine samples were obtained, drawn directly from the urinary bladder. Glucose concentration in the urine was measured by a colorimetric hexokinase glucose-6-phosphate dehydrogenase method (Glucose-HK kit, Spinreact, Spain).

### Histological analysis and morphometric parameters

Samples for histology were fixed in 10% formalin for 12 hours and embedded in paraffin with vacuum infiltration processor (Tissue-Tek® VIP™ 5 Jr, Sakura, USA). Specimens were cut with microtome Ergostar HM 200 (Microm, Germany) to 4 μm thick sections and stained using resorcin-fuchsin (Roth, Germany) and haematoxylin-eosin (Fisher Diagnostics, USA). The histological slides were examined under Olympus BX50 (Japan) light microscope.

Estimation of the internal diameter of the aorta was performed by measuring two inner diameters at right angle for each cross-section of the thoracic aorta. At least four different cross-sections of the aorta were analysed for each rat. Thickness of the medial layer of the aorta was determined in the thoracic aorta cross-sections by ten consecutive measurements in a systematic manner to evaluate all segments of the circumference of the aorta. At least four different cross-sections of aorta were analysed for each rat.

The staining intensity of elastin and collagen fibres in the media was evaluated by a subjective scale ranging from 0 to 3 (0 – no staining of fibres, 1 – poor staining of fibres, 2 – moderate staining of fibres, 3 – intensive staining of fibres). Evaluations were performed by two independent observers in a blinded fashion; the scores were summed and used for statistical analysis.

### Immunohistochemistry

Three-μm thick paraffin sections mounted on poly-L-lysine coated SuperFrost slides (Menzel-Gläser, Germany) were deparaffinised and rehydrated. Peroxidase activity was blocked by 0.6% H_2_O_2_ (Merck, Germany) in methanol (Merck, Germany). Then the sections were washed in tap water and in PBS (pH = 7.4; Gibco, Invitrogen, USA) for 10 min, treated with normal 1.5% goat serum (Gibco, Invitrogen Corporation, USA) for 20 min at room temperature and incubated with the primary antibody anti-carboxymethyl lysine (mouse monoclonal antibody [CML26], abcam, UK) diluted 1:50 overnight at 4°C in the humidity chamber. On the next day, the sections were incubated with the biotinylated horse anti-mouse secondary antibody for 30 min at room temperature. After a wash step the sections were incubated with the avidin-biotin peroxidase complex ELITE system (Vectastain Elite ABC Kit, Vector Laboratories Inc., Burlingame, USA) for 30 minutes. Peroxidatic activity was detected with 3,3′-Diaminobenzidine (Vector Laboratories Inc., USA) and the sections were counterstained with hemalaun, dehydrated and mounted with DPX (Fluka, Switzerland). The labeling was expressed on a subjective scale ranging from 0 to 4 (0 – no staining, 1 – weak staining, 2 – moderate staining, 3 - strong staining, 4 – very strong staining). Two independent observers in a blinded fashion performed the evaluation. The scores were summed and used for statistical analysis (Mann–Whitney U-test). Immunohistochemistry negative controls were performed by omitting primary antibody (mouse IgG was used in place of the primary antibody).

### Statistical analysis

All haemodynamic parameters were averaged into 5 mmHg MAP bins. Analysis was restricted to the MAP range of 60 to 185 mmHg. Rat S11 and S12 were merged to create one sample, due to lack of data in each rat. Rat S14 was removed due to lack of data. Second order polynomials were fitted using least squares regression to each individual rat data set, a second order polynomial being confirmed as a better fit than either linear or third order polynomial by Akaike’s “An Information Criterion (AIC)” (Table 1). The intercepts (*a*) and coefficients (*b*, *c*) were extracted from the polynomial model (Equation 1), and comparison between groups made by analysis of variance (ANOVA), using a post-hoc, Bonferroni corrected *t*-tests to ascertain differences.

(1) PWV=a+b×MAP+c×MAP2

(2) $$\mathrm{MAP}2\;=\;{\mathrm{MAP}}^{2}$$

**Table 1:** Model 1: linear; Model 2: second order polynomial; Model 3: third order polynomial fit

	**Df**	**AIC**
Model 1	5.00	970.37
Model 2	6.00	786.11
Model 3	7.00	787.55

A second order polynomial provides the best fit to the grouped data.

*Df*, degrees of freedom; *AIC*, Akaike information criterion.

For analysis of the data as a whole, one of two methods was employed. (1) The data was transformed into a linear form (Equation 2) and analysis of covariance (ANCOVA) was used if the assumption of equal slopes was maintained. (2) If the assumption of equal slopes was not maintained, robust analysis ANCOVA [20] was employed with comparison of groups at 60, 90, 120, 150, and 180 mmHg MAP and a 20% trimmed mean. The robust analysis ANCOVA accommodates non-linearity, heteroscedasticity, and unequal slopes. The assumption of equal slopes was tested by ANCOVA as in (Equation 1) with an interaction term between PWV and MAP, and PWV and MAP2. If either interaction was significant, showing that slopes were not equal, method (Equation 2) was employed.

Basic parameters and laboratory results are expressed as means ± standard deviation (SD). Differences between the groups were evaluated using the one-way ANOVA followed by Tukey’s *post-hoc* analysis for multiple comparisons of group means. Semi-quantitative data were compared by the Kruskal-Wallis one-way ANOVA followed by Mann–Whitney U test. Differences were considered to be statistically significant when P was <0.05. Statistical comparisons were performed with the Statistica software (version 8; StatSoft, USA) and with the software R (version 2.15.3 for Windows; The R Foundation for Statistical Computing, Austria).

## Results

### Basic and biochemical parameters

The initial body weights were similar in all groups (Table 2). In the course of the experiment, rats in both treated and untreated diabetic groups presented with abnormalities associated with persistent hyperglycaemia, i.e., hyperphagia, polydipsia, polyuria, and wasting of stored fat as evidenced by retarded weight gain. The final body weights were significantly lower in the diabetic groups compared to the control group, while no difference was observed between the treated and untreated diabetic groups. The ratio of heart weight to body weight as a surrogate index of cardiac hypertrophy was significantly increased in both diabetic groups, compared to the control group. Glucose concentration in the urine samples was significantly higher in both diabetic groups, compared to the control group. Treatment with telmisartan resulted in slight but statistically significant reduction in urinary glucose excretion (Table 2).

**Table 2:** Basic and laboratory parameters

**Group**	**Body weight (g)**		**Heart weight (mg)**	**Cardiac index (%)**	**Urine glucose (mmol/L)**
	**Before**	**After**			
Control	130 ± 10	408 ± 24	950 ± 90	2.32 ± 0.22	1.6 ± 0.9
Diabetes	135 ± 12	216 ± 59^*^	620 ± 130^*^	2.79 ± 0.25^¶^	511.6 ± 97.2^*#^
Diabetes + telmisartan	135 ± 14	254 ± 62^*^	670 ± 120^*^	2.67 ± 0.35^¶^	424.9 ± 63.5^*^

Body weight was assessed at the beginning and at the end of the experiment.

^¶^P <0.05 vs Control; ^*^P <0.001 vs Control; ^#^P <0.05 vs Diabetes + telmisartan.

Cardiac index = heart weight (mg)/body weight (g).

### Haemodynamic parameters

Prior to administration of vasoactive substances, resting systolic blood pressure (SBP), diastolic blood pressure (DBP), pulse pressure, MAP, heart rate (HR), PWV, and maximal change in blood pressure (dP/dt) were recorded (Table 3). There were no differences in blood pressures between the control and untreated diabetic group, but SBP, DBP, and MAP were significantly lower in the telmisartan-treated group. HR was decreased in both diabetic groups, and further reduced by treatment with telmisartan, most likely due to lower resting MAP. Peak dP/dt, a surrogate index of left ventricular systolic function, was significantly decreased in the untreated diabetic rats and partially attenuated by telmisartan, compared to the control rats. As a result of significantly lower resting MAP in the telmisartan group, PWV was also significantly lower in that group with no differences between the control and untreated diabetic group.

**Table 3:** Resting anaesthetised haemodynamic parameters obtained before the administration of vasoactive substances

**Group**	**SBP**	**DBP**	**MAP**	**PP**	**HR**	**Peak dP/dt**	**PWV**
	**(mmHg)**	**(mmHg)**	**(mmHg)**	**(mmHg)**	**(beats/min)**	**(mmHg/s)**	**(m/s)**
Control	155 ± 15^#^	113 ± 15^#^	130 ± 28^#^	41 ± 3	462 ± 46	4024 ± 617	4.5 ± 0.5^#^
Diabetes	142 ± 13^#^	105 ± 13^#^	124 ± 14^#^	38 ± 9	386 ± 48^*#^	2186 ± 198^*#^	4.6 ± 0.9^#^
Diabetes + telmisartan	110 ± 14	75 ± 14	92 ± 12	35 ± 5	321 ± 26^*^	3294 ± 183^*^	3.6 ± 0.3

*SBP*, systolic blood pressure; *DBP*, diastolic blood pressure; *MAP*, mean arterial pressure; *PP*, pulse pressure; *HR*, heart rate; dP/dt, maximal change in pressure over time; *PWV*, pulse wave velocity.

^*^P <0.01 vs Control; ^#^P <0.01 vs Diabetes + telmisartan.

**Figure 1:**
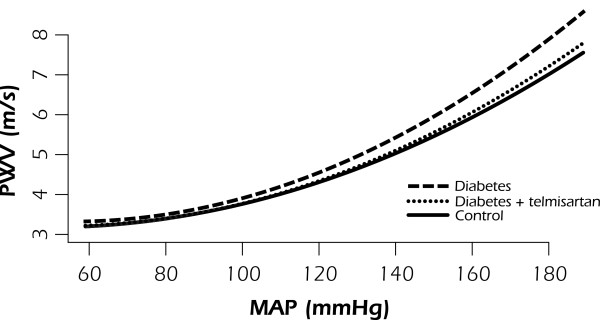
**A second order polynomial fitted on PWV-MAP curve, with the 95% confidence interval shaded.** PWV is significantly higher in the untreated diabetes group over the high pressure range, compared to the treated and control groups. No differences were observed among the treated and control groups across the full MAP range. MAP, mean arterial pressure.

The means and standard deviations of coefficients of the second order polynomial curve fits to individual rats in *PWV = a + b* × *MAP + c* × *MAP*^*2*^ are provided in Table 4 and polynomial curves are presented in Figure 1. ANOVA on the intercepts and coefficients is provided in Table 5. There was no significant difference in the intercept (*a*) between the groups, but there were significant differences in the coefficients (*b*) and (*c*) between the untreated diabetics and controls, but not among the other groups (Tables 6, 7, and 8).

**Table 4:** **Means and standard deviations of coefficients of the second order polynomial curve fits to individual rats in *PWV = a + b*** **×** ***MAP + c*** **×** ***MAP***^***2***^

**Group**	**a (mean ± SD)**	**b (mean ± SD)**	**c (mean ± SD)**
Control	3.8 ± 0.9	−0.02 ± 0.02	0.00023 ± 0.00009
Diabetes	6.0 ± 2.1	−0.07 ± 0.04*	0.00045 ± 0.00018*
Diabetes + telmisartan	5.1 ± 1.5	−0.05 ± 0.03	0.00038 ± 0.00014

*P <0.05, Bonferroni adjusted unpaired *t*-test. SD, standard deviation; MAP, mean arterial pressure.

**Table 5:** **Analysis of covariance with an interaction term between MAP and group, and MAP**^**2 **^**and group**

	**Df**	**Sum Sq**	**Mean Sq**	**F value**	**P value**
MAP	1	849.91	849.91	3953.44	<0.001
MAP2	1	47.35	47.35	220.27	<0.001
Group	2	13.41	6.70	31.18	<0.001
MAP:Group	2	5.58	2.79	12.97	<0.001
MAP2:Group	2	0.67	0.34	1.56	0.2
Residuals	571	122.75	0.21		

*MAP*, mean arterial pressure; *Df*, degrees of freedom; *Sum Sq*, sum of squares; *Mean Sq*, mean of squares.

Significant interaction between group and MAP indicates that the slopes of different groups are significantly different.

**Table 6:** **Robust method analysis of covariance, control (C) *vs *** **diabetes (D)**

**MAP**	**n1**	**n2**	**DIF**	**TEST**	**SE**	**CI.low**	**CI.hi**	**P value**	**crit.val**
60	23	10	−0.13	1.04	0.13	−0.63	0.37	0.03	3.95
70	42	21	−0.18	3.65	0.05	−0.34	−0.03	<0.001	3.07
80	45	28	−0.17	3.64	0.05	−0.31	−0.03	<0.001	3.08
90	46	32	−0.15	2.94	0.05	−0.31	0.01	0.01	3.05
100	45	36	−0.11	1.98	0.06	−0.28	0.06	0.05	3.00
110	45	38	−0.12	1.50	0.08	−0.35	0.12	0.14	3.02
120	45	39	−0.18	1.91	0.09	−0.46	0.10	0.06	3.00
130	45	38	−0.26	2.33	0.11	−0.58	0.07	0.02	2.99
140	45	39	−0.35	2.22	0.16	−0.82	0.12	0.03	3.01
150	45	38	−0.44	2.15	0.20	−1.05	0.18	0.04	3.03
160	45	38	−0.55	2.50	0.22	−1.21	0.12	0.02	3.04
170	44	35	−0.72	3.20	0.23	−1.41	−0.03	0.001	3.05

*MAP*, mean arterial pressure; *SE*, standard error; *CI*, confidence interval.

**Table 7:** Robust method analysis of covariance, diabetes (D) vs diabetes + telmisartan (DT)

**MAP**	**n1**	**n2**	**DIF**	**TEST**	**SE**	**CI.low**	**CI.hi**	**P value**	**crit.val**
60	10	21	0.12	0.98	0.12	−0.38	0.62	0.36	4.12
70	21	35	0.19	3.95	0.05	0.04	0.34	<0.001	3.10
80	28	40	0.21	4.51	0.05	0.07	0.36	<0.001	3.07
90	32	43	0.23	3.98	0.06	0.06	0.41	<0.001	3.01
100	36	45	0.16	2.08	0.08	−0.07	0.40	0.04	3.01
110	38	44	0.11	1.07	0.11	−0.20	0.43	0.29	2.99
120	39	42	0.16	1.43	0.11	−0.17	0.48	0.16	2.99
130	38	40	0.25	2.01	0.12	−0.12	0.62	0.05	3.00
140	39	40	0.36	2.13	0.17	−0.15	0.86	0.04	3.00
150	38	39	0.35	1.70	0.21	−0.27	0.98	0.10	3.02
160	38	33	0.53	2.37	0.22	−0.15	1.21	0.02	3.03
170	35	21	0.88	3.80	0.23	0.17	1.58	<0.001	3.06

*MAP*, mean arterial pressure; *SE*, standard error; *CI*, confidence interval.

**Table 8:** **Robust method analysis of covariance, diabetes + telmisartan (DT) *vs *** **control (C)**

**MAP**	**n1**	**n2**	**DIF**	**TEST**	**SE**	**CI.low**	**CI.hi**	**P value**	**crit.val**
60	23	21	−0.01	0.20	0.06	−0.20	0.18	0.85	3.12
70	42	35	0.01	0.15	0.05	−0.13	0.14	0.88	3.00
80	45	40	0.05	1.34	0.03	−0.06	0.15	0.19	2.99
90	46	43	0.08	1.62	0.05	−0.06	0.22	0.11	2.99
100	45	45	0.05	0.69	0.08	−0.18	0.28	0.49	3.02
110	45	44	−0.00	0.04	0.09	−0.29	0.28	0.97	3.02
120	45	42	−0.02	0.21	0.10	−0.32	0.27	0.83	3.00
130	45	40	−0.01	0.06	0.12	−0.37	0.36	0.95	3.00
140	45	40	0.01	0.05	0.13	−0.40	0.41	0.96	3.00
150	45	39	−0.09	0.56	0.15	−0.54	0.37	0.57	2.99
160	45	33	−0.02	0.11	0.16	−0.49	0.45	0.92	3.00
170	44	21	0.15	0.90	0.17	−0.37	0.68	0.37	3.06

*MAP*, mean arterial pressure; *SE*, standard error; *CI*, confidence interval.

Table 5 gives an ANCOVA with interaction terms for MAP and Group, and MAP^2^ and group. It shows that there was a significant interaction between MAP and group (P <0.001) but not MAP^2^ and group (P = 0.21). As there was a significant interaction term, and slopes therefore unequal, robust methods ANCOVA was used to test between group differences. This showed differences between untreated diabetes and control groups at all levels other than 60, 100, and 110 mmHg MAP (Table 6). Untreated diabetes and treated diabetes groups differed at all MAP levels other than 60, 110, 120, 130, and 150 mmHg (Table 7). Controls were not significantly different to treated animals at any MAP value (Table 8).

### Aortic wall morphology and histomorphometry

**Figure 2:**
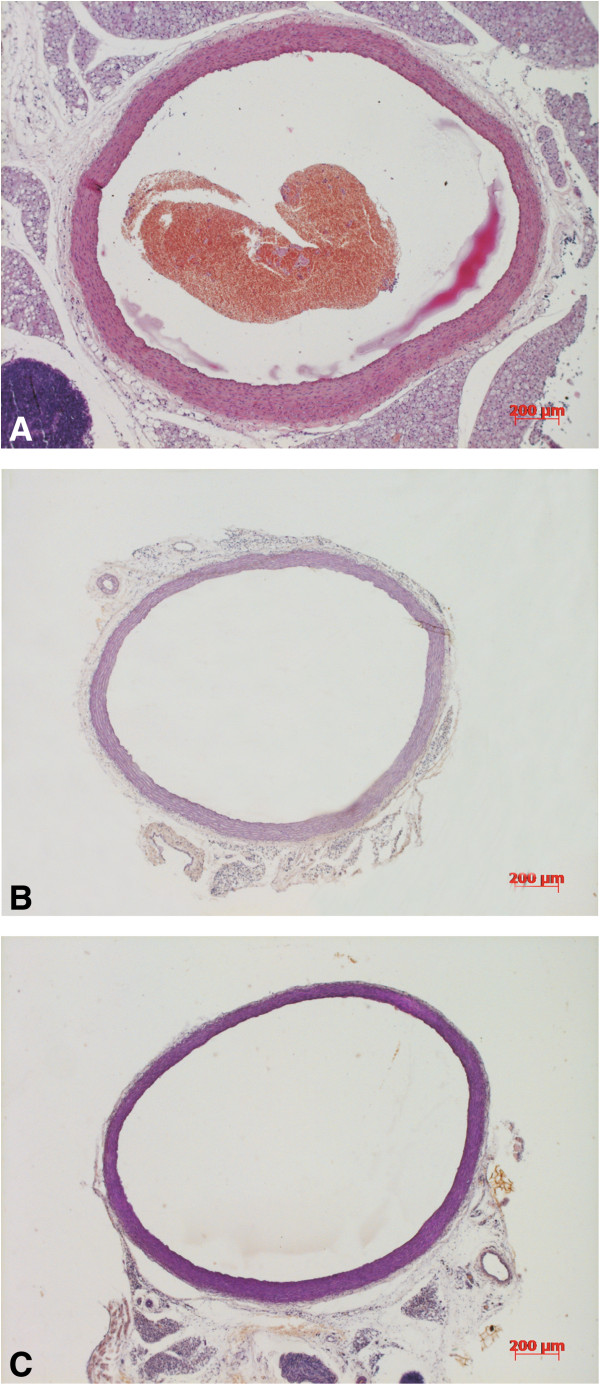
**Micrographs of the transverse sections in the control group (A), untreated diabetic group (B), and diabetes + telmisartan group (C).** Note the decreased width of the medial layer in both diabetic groups. Resorcin-fuchsin.

**Figure 3:**
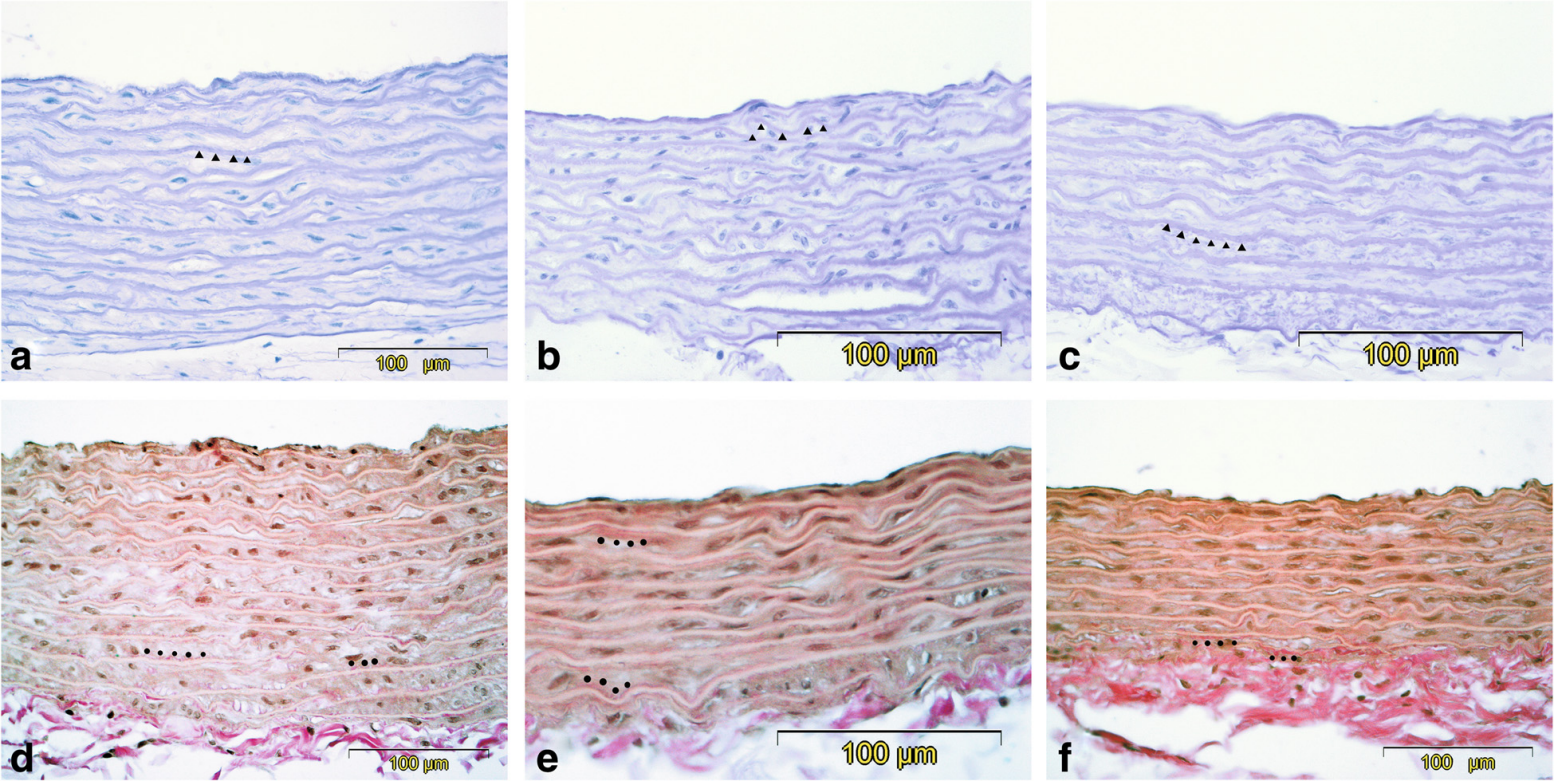
**Representative micrographs of the wall of the rat aortas from the control group (a and d), untreated diabetes group (b and e) and diabetes + telmisartan group (c and f).** Decreased thickness of the medial layer was noted in both untreated diabetes and diabetes + telmisartan group, while focal disorganisation of elastic lamellae were seen in untreated diabetes group **(b)**. Resorcin-fuchsin **(a-c)** and van Gieson **(d-e)**. ▲ elastin lamellae are stained violet (resorcin-fuchsin). ● collagen fibres are stained red (van Gieson).

The lumen diameter of the thoracic aorta was significantly smaller in the untreated diabetic group compared to the control and telmisartan groups (Table 9). The width of the medial layer was decreased both in the untreated diabetic and telmisartan-treated groups compared to the control group (Table 9, Figure 2). The staining intensity of medial elastic fibres in the untreated diabetic group was decreased compared to the control group, but in the telmisartan group the staining of elastic fibres did not differ significantly from the controls (Table 9). Furthermore, focal irregularities in the arrangement of elastic fibres were noted in the untreated diabetic group that were not observed in the telmisartan group (Figure 3). Even more pronounced differences were seen in the staining of collagen fibres in the medial layer as higher collagen concentrations were present in the untreated diabetes group compared to the control group (Table 9, Figure 3). This resulted in significantly lower medial ratio of elastin to collagen in the untreated diabetic group (Table 9). Although elastin to collagen ratio tended to increase in the telmisartan group it still remained lower than that in the control group (Table 9). No differences between groups were seen in the count of nuclei of smooth muscle cells (Table 9).

**Table 9:** Histomorphometric parameters of the thoracic aortas

**Parameter**	**Control**	**Diabetes**	**Diabetes + telmisartan**
Internal diameter of aorta (mm)	1.65 ± 0.18	1.23 ± 0.19^**¶**^	1.54 ± 0.22^#^
Width of media (μm)	117.39 ± 16.30	82.62 ± 9.32^**¶**^	87.68 ± 11.11^**¶**^
Elastic fibres in media (arbitrary units)	2.84 ± 0.23	2.54 ± 0.34^*^	2.65 ± 0.27
Collagen fibres in media (arbitrary units)	0.66 ± 0.19	1.43 ± 0.31^**¶**^	1.30 ± 0.10^**¶**^
Elastin/collagen ratio in media (%)	4.28 ± 0.57	1.79 ± 0.035^**¶**^	2.07 ± 0.50^*^
Smooth muscle cell nuclei	78.86 ± 15.01	94.10 ± 21.12	89.17 ± 24.90
CML (arbitrary units)	0.53 ± 0.08	2.28 ± 1.05^**¶**^	1.55 ± 0.80^**¶**^

Values are expressed as means ± SD. CML, *N*ϵ-(carboxymethyl) lysine.

^*^P <0.05 vs Control; ^**¶**^P <0.001 vs Control; ^#^P <0.05 vs Diabetes.

**Figure 4:**
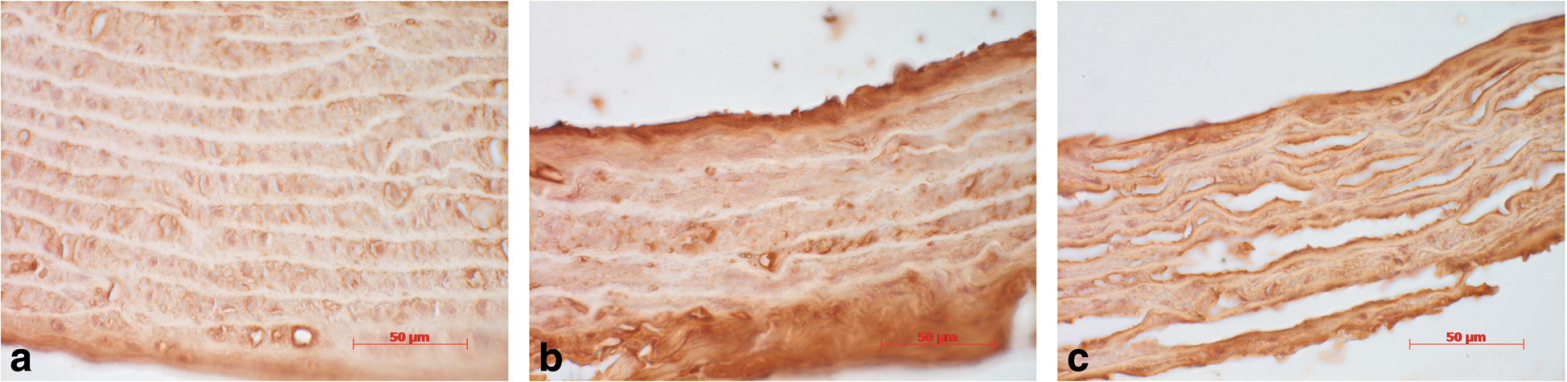
**Immunohistochemical localisation of *N*ϵ-(carboxymethyl) lysine in the rat aorta of control group (a), untreated diabetes group (b) and diabetes + telmisartan group (c).** Strong immunostaining (brown colour) was found in the aortas of diabetes group **(b)**, the immunostaining was moderate in the in diabetes + telmisartan group **(c)**, while in the control group the staining intensity was weak **(a)**. Diaminobenzidine + hemalaun.

Immunohistochemical detection of CML demonstrated strong immunostaining in the aortas of the untreated diabetes group with the strongest staining in the intima and adventitia. Staining was moderate in the telmisartan-treated group and weak in the control group (Figure 4, Table 9). Staining was not seen in the negative controls where the primary antibody was omitted.

## Discussion

In the present study, we have examined the association between structural (geometry and composition) and functional (stiffness, blood pressure) changes in the aortic wall and the protective effects of the ARB, telmisartan, in STZ-diabetic rats. We have demonstrated that treatment with telmisartan limits diabetes-induced aortic stiffening and improves the structural integrity of elastic fibres within the aortic wall.

Our finding of increased aortic wall stiffness following 10 weeks of untreated hyperglycaemia extends our previous study demonstrating that blood pressure-independent aortic stiffening is established early in the course of DM and is not associated with elevated resting BP [18]. In the current study, were able to reproduce similar DM-induced vascular changes in significantly younger animals (4 months *vs* 4 weeks at the induction of DM), thus eliminating the possible influence of aging on arterial remodelling.

Pharmacological modulation of BP allows characterisation of aortic stiffness, as measured by PWV, under pressure-independent conditions. This is important to consider, because BP exerts a great influence on PWV, and its physiological variations occur rapidly and frequently. Non-linear relationship between PWV and MAP was established by fitting a second-order polynomial function for the full range of experimentally controlled pressures. Across the low pressure range, PWV-MAP curves were similar between all groups, whereas at higher pressures, noticeable differences between the slopes of the curves were observed in different experimental groups. Steeper PWV-MAP slope in the untreated diabetic group most likely indicates increased aortic stiffness attributable to intrinsic modifications within the aortic wall independent of BP. Furthermore, the absence of elevated BP in untreated diabetic rats assessed under resting conditions gives additional support to the significance of underlying morphological abnormalities. Earlier studies have also shown that STZ-diabetic rats may be normotensive at the early stage of the disease [21], while impaired large artery properties may be detected as early as 8 weeks after induction of STZ-diabetes [22,23].

Functional properties of the aortic wall are determined by the content of two major wall constituents, elastin and collagen fibres, and their orderly arrangement. Elastin fibre network is known to mediate the load at low distending pressures, while collagen fibres are gradually recruited with increasing pressure [24]. In our study, significant reduction in the aortic content of elastin was observed in the untreated diabetic group, paralleled by an increase in collagen content, resulting in lower ratio of elastin to collagen. These findings are concordant with the profile of aortic stiffness in the untreated diabetic group. When BP increases, loss of elastin and increase in collagen content causes premature recruitment of stiffer collagen fibres as demonstrated by higher isobaric PWV across high BP range. Studies on the biomechanical properties of elastin and collagen have shown that when elastase-digested arteries are stretched, collagen fibres are more rapidly engaged, in contrast to smooth and gradual recruitment in normal elastic arteries [25,26]. The transition point from recruitment of elastin to collagen fibres could not be mathematically determined in our study, but seems to be around 110–120 mmHg, which is in agreement with an earlier report by Armentano *et al.*[24].

Diabetes-induced arterial wall remodelling is a well-established complication and characterised by thickening of intimal and medial layers. Increased carotid artery intima-media thickness has frequently been reported in patients with type 1 DM [27,28] and is considered an independent CV risk factor [29]. In this regard, our findings of reduced width of aortic media in the untreated diabetic group are at variance with observations from human studies. However, experimental studies with different durations of STZ-diabetes have shown that at an early stage, the width of the aortic medial layer may be decreased [30,31], and gradually increase with the ongoing disease due to vascular smooth muscle cell proliferation [32]. The structural alterations occurring during the course of DM may involve differential transcriptional regulation of matrix metalloproteinases [33] and transforming growth factor-beta [34,35]. The accelerated formation of AGEs driven by sustained hyperglycaemia also plays a critical role in the pathogenesis of diabetic macrovascular complications [34,36]. Indeed, we have shown that the accumulation of *N*ϵ-(carboxymethyl) lysine (CML), a major antigenic structure among a variety of AGEs [37], in the aortic wall is significantly enhanced in untreated DM [36] which was also the case in the current study. Similarly to our previous experiment we observed that CML was localised in all layers of the aorta with prominent levels in the intima and adventitia.

Telmisartan is an ARB with significant protective effects against tissue remodelling, in addition to equipotent blood pressure reducing effects, compared to other RAS inhibitors, captopril or losartan [38]. Telmisartan has been reported to act as a partial agonist of peroxisome proliferator-activated receptor-γ (PPAR-γ) [39], which is involved in the regulation of carbohydrate and lipid metabolism [40]. Furthermore, there is increasing evidence that PPAR-γ agonists exert anti-inflammatory, antioxidative, and antiproliferative effects on the vascular wall [40,41]. We report that treatment of STZ-diabetic rats with telmisartan for 10 weeks was able to attenuate the development of DM-induced aortic stiffening in the context of improved structural properties of the aortic wall. Specifically, telmisartan preserved the amount of elastin within the medial layer of the aorta, and maintained the normal organisation of elastin network. These effects were also associated with a modest, but insignificant, increase in the ratio of elastin to collagen. The anti-fibrotic effects of telmisartan were evident despite no effect on glucose control or body weight. To our best of knowledge, the current study is the first to report that the ARB telmisartan modulates DM-induced aortic stiffening and remodelling independently on BP reduction. Previous studies have demonstrated that, in addition to direct inhibition of the pro-fibrotic effects of Ang II, treatment with ARBs may have indirect pressure-independent protective effects by interfering with AGE formation via antioxidative mechanisms [42]. We found that treatment with telmisartan had a minor effect on reducing the accumulation of CML in the aortic wall that may have been limited by the pathological impact of hyperglycaemia. Miyata and co-workers [42] demonstrated *in vitro* that ARBs are effective against AGE formation that has been confirmed in experimental studies with murine type 2 DM models; however, to our best of knowledge, there is a lack of interventional studies showing similar results in experimental type 1 DM models.

In summary, we have shown that the ARB telmisartan prevents aortic stiffening associated with untreated STZ-diabetes in the context of improved structural properties of the aortic wall such as preservation of the concentration and organisation of elastin network. The results from our study provide further evidence that increased aortic stiffness is an early phenomenon in the pathogenesis of DM and its complications that is attributable to intrinsic modifications within the aortic wall, and that the inhibition of RAS has a specific role in the prevention of early DM-induced vascular damage beyond BP lowering effects.

## Competing interests

The authors declare that they have no competing interests.

## Authors’ contributions

ES performed the experiments, participated in performing the statistical analysis, and drafted the manuscript. MB and APA performed statistical analysis, participated in the study design and coordination, and helped to draft the manuscript. ES, PK, JK, MZ, JE conceived of the study, participated in its design and coordination, and helped to draft the manuscript. MA and AA performed the histological analyses and helped revising the manuscript. All authors have read and approved the final manuscript.
